# Chronic Shoulder Dislocation on the Unaffected Side of a Patient With Hemiparesis

**DOI:** 10.7759/cureus.34358

**Published:** 2023-01-29

**Authors:** Tomohiko Sano

**Affiliations:** 1 Department of Orthopaedics, Owase General Hospital, Owase, JPN

**Keywords:** weight-bearing shoulder, latarjet's procedure, open reduction, hemiparesis, chronic anterior shoulder dislocation

## Abstract

Chronic shoulder dislocation has been noted to be difficult to cure due to concomitant injuries of the soft tissue, articular cartilage, and bone. The present study reports a rare case of a patient with hemiparesis suffering chronic shoulder dislocation on the unaffected side. The patient was a 68-year-old female. She developed left hemiparesis due to cerebral bleeding at 36 years of age. Her right shoulder was dislocated for three months. A computed tomography scan and magnetic resonance imaging (MRI) showed a significant anterior glenoid defect, and the subscapularis, supraspinatus, and infraspinatus were atrophic. An open reduction with transfer of the coracoid, Latarjet's method was performed. The rotator cuffs were simultaneously repaired using McLaughlin’s method. The glenohumeral joint was temporarily fixed with Kirschner wires for three weeks. There was no redislocation during the 50-month follow-up period. Even though radiographs noted progression of osteoarthritis in the glenohumeral joint, the patient reacquired shoulder function for ativities of daily living including weight-bearing ability.

## Introduction

Chronic dislocation is defined as the shoulder remaining dislocated for three weeks or more [1]. A chronic anterior shoulder dislocation (CASD), although uncommon, is difficult to cure even in the current era due to a combination of injuries to the articular cartilage, glenoid or humeral bone, and soft tissue such as rotator cuff or labrum [2,3]. Several studies have recommended surgery to treat CASD since conservative treatment tends to result in severe functional limitations and pain [3,4]. Surgical options, including open reduction, and temporary glenohumeral fixation with Kirshner wires, Bankart repair, remplissage, coracoid transfer, bone-grafting, and arthroplasty have all been reported [2,4-7].

Patients with complete hemiplegia generally use the unaffected upper extremity as the weight-bearing limb during activities of daily living. Surgery on the weight-bearing shoulder is a challenge since the abnormal stress leads to a higher incidence of shoulder disorders such as tendinitis, rotator cuff tear, and arthritis [8-10]. The specific pathology and previous overuse of the joint should be considered when determining the appropriate therapeutic strategy for the weight-bearing shoulder [11].

## Case presentation

**Figure 1:**
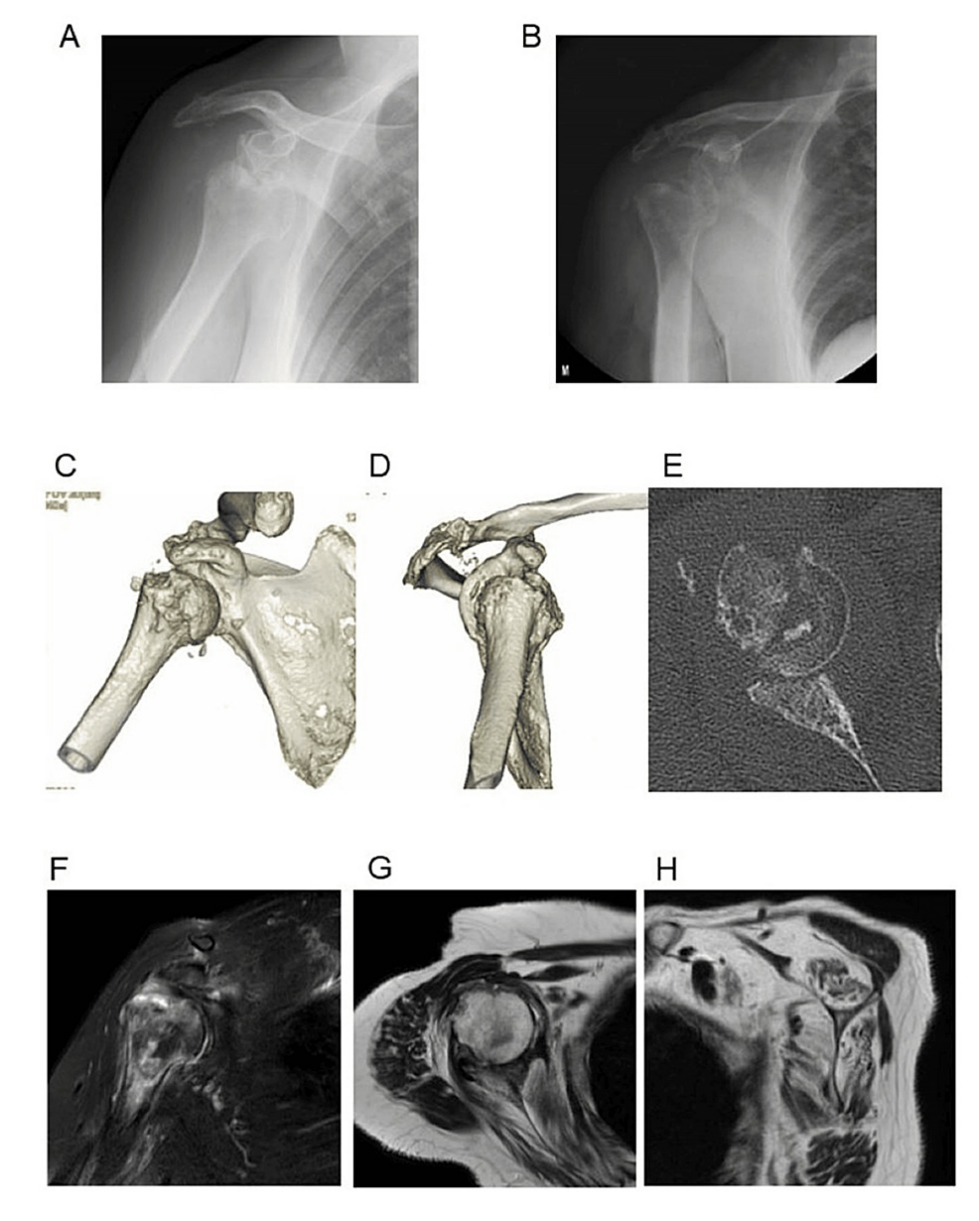
The preoperative images. A) Shoulder X-ray at the initial visit to our hospital. B) X-ray fluoroscopy after the attempted closed reduction. C-E) CT scans showing a large Hill-Sachs lesion and defect of the anterior glenoid surface. F) T2-weighted fat suppression image on coronal MRI showing a broad bone bruise in the humeral head. G, H) T2-weighted MRI showing severe muscle atrophy and fatty infiltration of the subscapularis, supraspinatus, and infraspinatus, indicating chronic significant rotator cuff tear.

**Figure 2:**
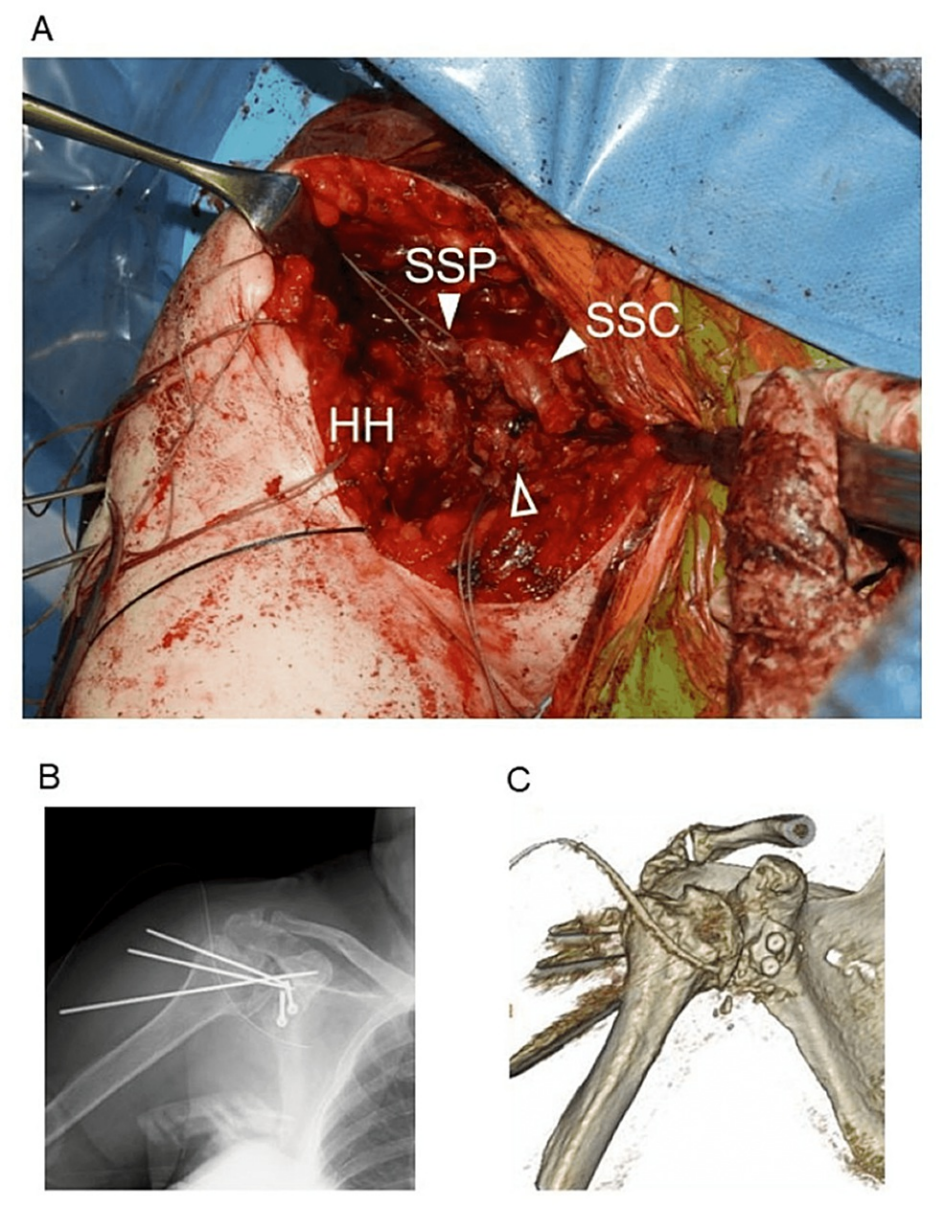
The perioperative images. A) The intraoperative photograph immediately following reduction of the humeral head and transfer of the coracoid to the glenoid. White arrowheads indicate full-thickness tear of the subscapularis and supraspinatus. The white outlined arrowhead indicates the transferred coracoid. SSP: supraspinatus, SSC: subscapularis, HH: humeral head. B, C) Postoperative X-ray and CT scan.

**Figure 3:**
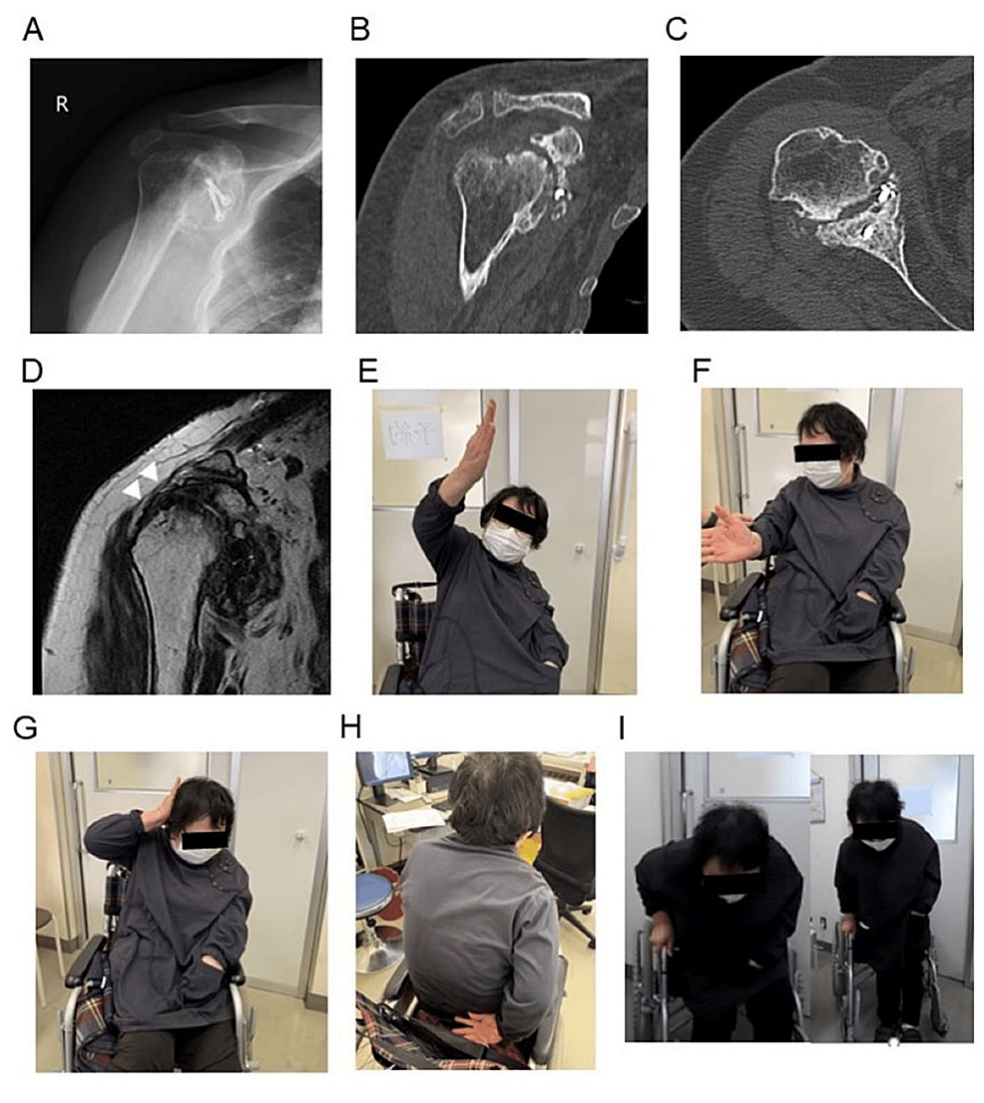
Fifty months after surgery. A) Radiograph showing progression of the osteoarthritis. B, C) CT scans showing reconstruction of the glenoid surface by the transferred coracoid. D) White arrowheads indicate integrity of the repaired supraspinatus tendon on T2-weighted MRI. E) Flexion. F) External rotation. G) The patient can touch the back of her head with the elbow back. H) Internal rotation. I) The patient can stand up by herself.

The patient was a 68-year-old woman with complete hemiplegia of her left side due to a cerebral hemorrhage at 36 years of age. She was functionally independent and could stand up and walk with a cane, using her right arm as a weight-bearing limb. Shortly after falling down, she was diagnosed with a right shoulder anterior dislocation that was reduced under general anesthesia at a hospital near her home. Three days after the reduction, X-ray imaging at the same hospital showed that the shoulder had dislocated again. However, the dislocation was not treated due to difficulty with the initial reduction. Three months following the initial dislocation, she presented to our hospital due to continuous pain and difficulty standing up by herself. Despite the aid of X-ray fluoroscopy and a scalene block, a closed reduction could not be accomplished (Figure 1A, 1B). A computed tomography (CT) scan showed a large Hill-Sachs lesion with approximately a 40% loss of the glenoid surface anteriorly (Figure 1C-1E). Magnetic resonance imaging (MRI) revealed atrophy of the subscapularis, supraspinatus, and infraspinatus, indicating that these rotator cuffs had torn (Figure 1F-1H). Her shoulder's active range of motion at the first visit to our hospital was 20° in flexion, -20° in external rotation, and the hip level in internal rotation. Constant and Rowe scores are 4 and 0 points, respectively. To reduce the dislocation and restore stability, an open reduction with a deltoid-pectoral incision and transfer of the coracoid to the glenoid using Latarjet's method was performed. Intraoperatively, progressive osteoarthritis of the glenohumeral joint was noted, and the subscapularis, supraspinatus, and infraspinatus tendons were noted to be completely torn (Figure 2A). Circumferential capsular release was required to reduce the dislocated humeral head due to soft tissue contracture. The rotator cuff tears were repaired by McLaughlin's method. Finally, the glenohumeral joint was fixed with Kirschner wires to maintain the reduction (Figure 2B, 2C). The Kirschner wires were removed three weeks after the operation and mild passive range of motion (ROM) exercises were started. Active ROM exercise and weight-bearing were started at six and eight weeks, respectively. There has been no re-dislocation during the 50-month follow-up period. While X-ray imaging noted progression of osteoarthrosis of the glenohumeral joint (Figure 3A), a CT scan noted the transferred coracoid reconstructed the defect within the anterior rim of the glenoid (Figure 3B, 3C). MRI demonstrated integrity of the repaired rotator cuff tear (Figure 3D). Her shoulder's active range of motion is currently 120° in flexion, 30° in external rotation, hand behind the head with elbow back, and the fifth lumber spine in internal rotation (Figure 3E-3H). Constant and Rowe scores are 53 and 65 points, respectively. She can stand up and walk with a cane by herself without any pain.

## Discussion

Several head-preserving procedures have been reported as surgical treatment for CASD. Rouhani et al. performed an open reduction and Bankart repair for eight patients with an unengaged type of CASD [12]. It resulted in an average of 86 points on Rowe and Zarin's score, although two shoulders remained subluxed. Liu et al. reported that open reduction and coracoid transfer procedures resulted in an average score of 59 and 62 points in the Constant and American Shoulder and Elbow scales, respectively [2]. The overall redislocation and subluxation rate was 48% (12/25). The rate was 0% (0/5) for the subscapularis splitting group, 53% (8/15) for the subscapularis tenotomy and repair group, and 80% (4/5) for the humeral head replacement group. Akinci et al. used Kirshner-wire from the acromion to the humerus in four cases, and from the humerus to the glenoid in six cases, for temporal stabilization following open reduction of the dislocation [5]. They reported no redislocation or complications. Flatow et al. employed open reduction and replacement arthroplasty combined with coracoid transfer for CASD with a glenoid defect [7]. They reported excellent or satisfactory results in eight of nine cases.

In determining the appropriate surgical option in this case, consideration that she had used her dislocated shoulder as a weight-bearing joint in her daily life for a number of years was necessary. Initially, total shoulder arthroplasty (TSA) or reverse shoulder arthroplasty (RSA) was considered since there were significant bony defects of the glenoid, a large Hill-Sachs lesion in the humeral head, and a chronic large rotator cuff tear were noted on preoperative CT and MRI. Garreau De Loubresse et al. reviewed four cases of TSA and a case of hemiarthroplasty for osteoarthritis or avascular necrosis of the weight-bearing shoulder [13]. They reported the necessity for an early revision following a loose glenoid screw and a cemented glenoid implant migration at 30 months postoperatively. Patel et al. stated that the implant was not designed to accommodate weight-bearing loads following an RSA [11]. Additionally, the long-term prognosis of arthroplasty for a weight-bearing shoulder had not been established at the time of the operation. For these reasons, joint preserving surgery was employed as the first choice. To avoid early postoperative loosening and redislocation, temporary glenohumeral joint fixation with Kirshner wires was also performed. As a result, she regained her ability to perform activities of daily living, and there was no redislocation during the 50-month follow-up period. Even though osteoarthritis in the glenohumeral joint progressed, the transferred coracoid reconstructed the defective anterior rim of the glenoid. Recently, satisfactory short-term results of RSA for weight-bearing shoulders were reported [14]. However, it appears that the surgical treatment provided in this case was meaningful even if the patient’s shoulder needs future replacement arthroplasty.

## Conclusions

The present study reports the clinical course of surgical treatment of a chronic anterior shoulder dislocation in the unaffected side of a patient with hemiparesis. To the best of our knowledge, no similar case has ever been reported. Open reduction with circumferential detachment of the joint capsule was needed to reduce the humeral head which had been dislocated for three months. The significant anterior glenoid defect was reconstructed with a coracoid transfer, Latarjet's procedure, and the rotator cuff tears were repaired utilizing McLaughlin's procedure. The temporary glenohumeral joint fixation with Kirshner wires was effective in preventing postoperative loosening. There has been no redislocation in the 50 months following surgery. Even though radiographs noted progression of osteoarthritis in the glenohumeral joint, the patient reacquired shoulder function for activities of daily living including the ability to bear weight.
